# Sirolimus-Based Immunosuppression Is Associated with Decreased Incidence of Post-Transplant Lymphoproliferative Disorder after Heart Transplantation: A Double-Center Study

**DOI:** 10.3390/jcm11020322

**Published:** 2022-01-10

**Authors:** Rabea Asleh, Darko Vucicevic, Tanya M. Petterson, Walter K. Kremers, Naveen L. Pereira, Richard C. Daly, Brooks S. Edwards, D. Eric Steidley, Robert L. Scott, Sudhir S. Kushwaha

**Affiliations:** 1 Department of Cardiovascular Diseases and Health Sciences Research and the William J von Liebig Center for Transplantation and Clinical Regeneration, Mayo Clinic, Rochester, MN 55905, USA; kremers.walter@mayo.edu (W.K.K.); pereira.naveen@mayo.edu (N.L.P.); Daly.richard@mayo.edu (R.C.D.); edwards.brooks@mayo.edu (B.S.E.); kushwaha.sudhir@mayo.edu (S.S.K.); 2 Heart Institute, Hadassah University Medical Center, Faculty of Medicine, Hebrew University of Jerusalem, Jerusalem 9112001, Israel; 3 Department of Cardiology, David Geffen School of Medicine at UCLA, Los Angeles, CA 90095, USA; darkovucicevic@yahoo.com; 4 Division of Biomedical Statistics and Informatics, Department of Health Sciences Research, Mayo Clinic, Rochester, MN 55905, USA; Petterson.tanya@mayo.edu; 5 Department of Cardiovascular Diseases, Mayo Clinic Arizona, Phoenix, AZ 85054, USA; steidley.d@mayo.edu (D.E.S.); scott.robert@mayo.edu (R.L.S.)

**Keywords:** mTOR inhibitors, sirolimus, post-transplant lymphoproliferative disorder, heart transplantation, immunosuppression

## Abstract

Mammalian target of rapamycin (mTOR) inhibitors have been shown to reduce proliferation of lymphoid cells; thus, their use for immunosuppression after heart transplantation (HT) may reduce post-transplant lymphoproliferative disorder (PTLD) risk. This study sought to investigate whether the sirolimus (SRL)-based immunosuppression regimen is associated with a decreased risk of PTLD compared with the calcineurin inhibitor (CNI)-based regimen in HT recipients. We retrospectively analyzed 590 patients who received HTs at two large institutions between 1 June 1988 and 31 December 2014. Cox proportional-hazard modeling was used to examine the association between type of primary immunosuppression and PTLD after adjustment for potential confounders, including Epstein–Barr virus (EBV) status, type of induction therapy, and rejection. Conversion from CNI to SRL as primary immunosuppression occurred in 249 patients (42.2%). During a median follow-up of 6.3 years, 30 patients developed PTLD (5.1%). In a univariate analysis, EBV mismatch was strongly associated with increased risk of PTLD (HR 10.0, 95% CI: 3.8–26.6; *p* < 0.001), and conversion to SRL was found to be protective against development of PTLD (HR 0.19, 95% CI: 0.04–0.80; *p* = 0.02). In a multivariable model and after adjusting for EBV mismatch, conversion to SRL remained protective against risk of PTLD compared with continued CNI use (HR 0.12, 95% CI: 0.03–0.55; *p* = 0.006). In conclusion, SRL-based immunosuppression is associated with lower incidence of PTLD after HT. These findings provide evidence of a benefit from conversion to SRL as maintenance therapy for mitigating the risk of PTLD, particularly among patients at high PTLD risk.

## 1. Introduction

Post-transplant lymphoproliferative disorder (PTLD) presents a group of heterogeneous diseases characterized by uncontrolled lymphoid cell proliferation as a consequence of immunosuppression after solid organ and hematopoietic stem cell transplantation [1,2]. PTLD can clinically vary from benign lymphoid hyperplasia to aggressive anaplastic or diffuse large B-cell lymphomas. PTLD development is related to induced suppression of T-cell immunity, with the incidence of non-Hodgkin lymphoma being 25- to 100-fold higher in transplant recipients as compared to matched subjects from the general population [3]. The incidence of PTLD is typically higher after transplantation of solid organs that require high-intensity immunosuppressive agents, such as in lung and heart transplantation (HT) [1,4].

The mammalian target of rapamycin (mTOR) pathway is involved in controlling cell growth, metabolism, and immune cell activation in response to nutrients, growth factors, and pathogens [5]. Sirolimus (SRL) and its derivative, everolimus, are mTOR inhibitors that exert antiproliferative properties; thus, both agents are increasingly used for immunosuppression and treatment of specific types of malignancies, as well as in drug-eluting stents for delaying coronary artery restenosis [5,6]. Among HT recipients, substitution of calcineurin inhibitor (CNI) with SRL as primary immunosuppression has been shown to attenuate cardiac allograft vasculopathy (CAV) and improve clinical outcomes [7,8]. Moreover, SRL has favorable effects on renal function and it has been increasingly used as a management approach for minimizing CNI-induced nephrotoxicity after HT [9,10].

Small observational studies have shown that SRL use in transplant recipients who develop non-metastatic malignancies results in regression of these tumors [11]. Moreover, SRL has been shown to inhibit growth and proliferation of abnormal lymphoid cells in both in vitro and in vivo PTLD models [12,13]. Several case reports and series have suggested a potential benefit from the use of mTOR inhibitors on risk of PTLD and disease progression [14,15]. We have recently demonstrated in a single institutional study that conversion to SRL was associated with significantly decreased risk of overall de novo malignancies post-HT, including the risk of PTLD when analyzed as a secondary outcome [16]. However, the decreased risk of PTLD with long SRL use has not been validated in a larger multicenter HT population.

The aim of this study was primarily to investigate the effect of conversion to SRL on risk of PTLD compared with a continued CNI-based regimen in a large cohort of HT recipients at two centers with large volumes of patients undergoing conversion to SRL with complete withdrawal of CNI therapy.

## 2. Methods

### 2.1. Data Source

This was a nonrandomized, retrospective, two-center study approved by the Mayo Clinic Institutional Review Board, with the same entry criteria included at the two Mayo Clinic institutions (ethics committee approval code: 16-006949). The charts of all patients who underwent HTs at Mayo Clinic in Rochester and Mayo Clinic in Arizona between 1 June 1988 and 31 December 2014 were retrospectively reviewed. Patients who were less than 18 years old or did not survive the index hospitalization were excluded from the study. Patients who had more than one HT were followed until the time of their second HT. The diagnosis of PTLD was confirmed on histopathology for all events. Data regarding demographics, induction and maintenance immunosuppression, rejection, incidence of PTLD, and Epstein–Barr virus (EBV) status were all abstracted. For patients diagnosed with PTLD, data on time from HT to diagnosis, histopathologic features, treatment, and outcomes were all collected for each patient. Patients who denied research authorization at Mayo Clinic in Rochester were excluded from data analysis in accordance with the Minnesota law. HT recipients were considered to be EBV mismatches if the donor was positive and the recipient had negative EBV serology at the time of HT. EBV serology status was considered “missing” if the donor had positive EBV serology and the recipient’s EBV serology information was missing; if the recipient had negative EBV serology and the donor’s EBV serology information was missing; or if both the recipient’s and the donor’s EBV serology information was missing.

### 2.2. Immunosuppression

The majority of patients at both institutions received induction therapy with low-dose muromonab-CD3 (OKT3) or antithymocyte globulin (ATG) as part of a standard induction protocol and a three-drug maintenance immunosuppressive regimen consisting of a CNI (cyclosporine A or tacrolimus), an antimetabolite agent (mycophenolate mofetil (MMF) or azathioprine), and tapering doses of prednisone post-HT. According to our protocol [16], rabbit ATG (1.5 mg/kg) was used at the time of HT and continued based on CD4 and CD8 T-cell counts until tacrolimus was in the goal range (10–14 ng/mL), in addition to MMF (1000–1500 mg twice daily) and steroid therapy. In the old HT era, OKT3 (doses based on the CD3 T-cell subset counts) was used for induction therapy and cyclosporine, azathioprine (1–3 mg/kg), and steroids were used as maintenance immunosuppression. Patients who were considered to be at lower risk of rejection did not receive induction at the discretion of the transplant cardiologist. All patients were initially placed on CNI-based immunosuppression. At Mayo Clinic Arizona, HT recipients were converted to SRL-based maintenance immunosuppression if they had evidence of renal dysfunction or findings suggestive of advanced CAV. At Mayo Clinic Rochester, the reasons for conversion to SRL varied according to the period of conversion. Until July 2006, most patients were converted to SRL due to impaired renal function secondary to CNI or advanced CAV or due to intolerance of CNI therapy. Since July 2006, a routine conversion protocol from CNI to SRL was introduced at approximately 6 months after HT regardless of patients’ baseline kidney function or CAV grade prior to the conversion process. According to protocol [7,17], patients in stable condition, without evidence of rejection and on antimetabolite and low-dose steroid therapy, received gradually increasing doses of SRL to achieve plasma levels of 10–14 ng/mL, and once SRL target levels were achieved, CNI dose was gradually reduced until complete withdrawal of CNI therapy. The dose of secondary immunosuppression, MMF or azathioprine, as well as the dose of prednisone, remained unchanged during the conversion process. Biopsy was generally repeated two weeks after conversion and a reduced dose of CNI was reintroduced if biopsy was positive for rejection, with a second attempt to withdraw CNI therapy later if rejection subsided.

### 2.3. Biopsies

Routine endomyocardial biopsies were performed according to our previously described institutional protocols [7,17]. Rejection episodes were classified according to the 2005 International Society of Heart and Lung Transplantation (ISHLT) consensus report [18]. Grade 2R and 3R acute cellular rejection and any antibody-mediated rejection (AMR) were considered significant and required augmentation of immunosuppression therapy as per protocol.

### 2.4. Statistical Analysis

The primary outcome was development of a PTLD event following HT. Descriptive statistics were used to informally describe baseline variables, using mean and standard deviation (SD) or median with the first and third quartiles (Q1, Q3) if data were heavily skewed for continuous variables and using percentages for categorical variables. Gender, age at HT, multiorgan transplant (yes/no), EBV mismatch (yes/no), and induction regimen were considered baseline variables. Missing EBV serology status was assessed, adjusting for EBV mismatch (both compared to no mismatch). Induction therapy was assumed to occur at the time of HT (time 0). Survival free of PTLD was estimated using the Kaplan–Meier product limit method, censoring for death. Date of HT was the index date (time 0). Data were censored at the earliest at death, date of last follow-up, or 31 December 2014. Censoring was assumed to be uninformative (not related to the probability of PTLD). The relationship between independent variables was examined using the Cox proportional-hazards model. We used stepwise Cox conditional regression with alpha ≤0.05 to enter and leave when developing a final model; adjusted Cox proportional-hazards models were also examined. Continuous variables were assessed for non-linearity using Martingale residuals and the proportional-hazard assumption was assessed using scaled Schoenfeld residuals. SRL was considered a time-dependent variable because it was started subsequent to HT. To test the association of SRL with PTLD, an indicator variable was set to “1” at the date of conversion to SRL and back to “0” if the patient was taken off SRL. Date of first rejection was also considered a time-dependent variable for analysis of association with PTLD; at date of first rejection, the variable “any rejection” was set to “1” and left as “1” regardless of any additional rejections. Time-dependent variables were analyzed using the start/stop count methodology described by Aalen et al. [19].

Standardized morbidity ratios were calculated using the person-years methodology. Counts of PTLD occurring while on or off of SRL were categorized in intervals from time of transplant as follows: 0 to 1 month, 1 to 6 months, 6 to 12 months, 1 to 2 years, 2 to 5 years, 5 to 10 years, and greater than 10 years. Patients on SRL were counted as on SRL throughout each interval after they began until such time as they developed PTLD, stopped treatment, were lost to follow-up, were censored on 31 December 2014, or died. For those patients not on SRL at the start of a given interval, but who began during the interval, person years not on SRL were tallied until they received treatment, at which time person years on SRL were tallied. Similarly, once off SRL or for those never treated, person years were tallied until patients developed PTLD, were lost to follow-up, were censored on 31 December 2014, or died. All statistical analysis was done using SAS, version 9.4 (SAS Institute Inc. Cary, NC, USA), or R, version 3.4.2.

## 3. Results

### 3.1. Patient Characteristics

**Figure 1:**
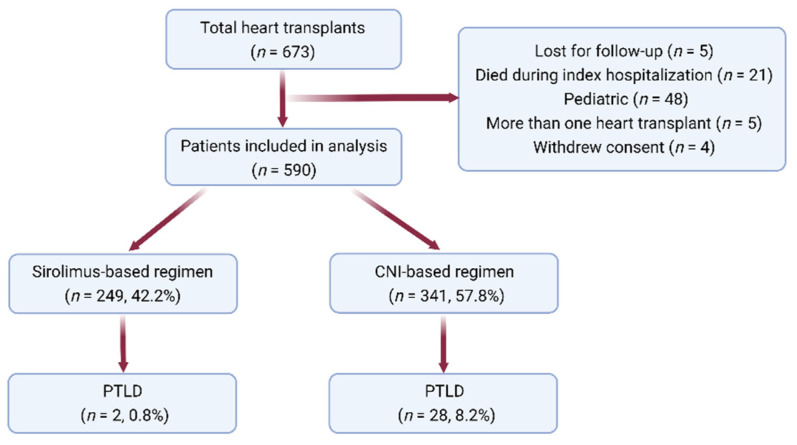
Flowsheet of the study. CNI, calcineurin inhibitor; PTLD, post-transplant lymphoproliferative disorder.

A total of 668 patients underwent HTs at Mayo Clinic in Rochester or in Arizona during the study period. After applying our exclusion criteria, we identified 590 patients who were included in the analysis. Data are presented in Figure 1. For the 590 patients studied, the median observed follow-up was 6.3 years (range 0.2–26.0 years), during which time 30 patients (5.1%) developed PTLD. The probability of PTLD was 0.5%, 0.8%, 1.4%, 2.6%, and 7.8% at 90 and 180 days and at 1, 5, and 10 years, respectively. Median (Q1, Q3) of the observed time to the development of PTLD was 4.4 (0.9, 7.5) years. Baseline characteristics of the PTLD and non-PTLD groups are presented in Table 1.

The recipient EBV status was available for 419 patients. Eighteen patients were identified as having an EBV mismatch (4.3%). Among 30 patients who developed PTLD, EBV status was available for 16 patients, of whom mismatch was documented in 5 (31.3%) as compared to 13 of the remaining 403 (3.2%) with an available EBV status but who had not developed PTLD by the date of censor. Induction therapy was performed for the majority of patients. Among the 433 patients for whom induction information was known, induction included low-dose OKT3 (in 48.5% of patients) or anti-thymoglobulin (ATG) (in 44.3%), while the remaining patients (7.2%) did not receive any type of induction therapy. Of the 590 HT patients studied, 187 (32%) developed at least one episode of clinically significant acute allograft rejection (2R grade or higher for cellular rejection or any grade of AMR). The probability of rejection was 17%, 21%, 25%, 28%, and 32% at 90 and 180 days and at 1, 2, and 5 years, respectively. Median (Q1, Q3) observed time to rejection was 3.4 years (0.7, 8.2). A total of 12 of the 187 patients developed acute rejection before the diagnosis of PTLD. Two patients developed acute rejection after a diagnosis of PTLD. The remaining 173 with rejection had no diagnosis of PTLD.

### 3.2. SRL-Based Immunosuppression as an Independent Predictor of PTLD

During follow-up after HT, 249 patients (42.2%) were converted to SRL as primary immunosuppression. The probability of conversion was 3%, 11%, 21%, 30%, and 39.5% at 90 and 180 days and at 1, 2, and 5 years, respectively. In the entire group, the median (Q1, Q3) observed time of conversion was 3.4 years (1.0, 7.7). The most common reasons for transition to SRL were our institutional protocol (*n* = 88; 35.3%), CNI-induced kidney toxicity (*n* = 81; 32.5%), development of advanced CAV (*n* = 45; 18.1%), severe CNI-associated side effects (*n* = 17; 6.8%), and recurrent allograft rejection (*n* = 16, 6.4%). Of the 249 patients converted to SRL, 2 patients developed a PTLD event, and none of the remaining 247 patients were diagnosed with PTLD during follow-up. Twenty-eight patients who developed PTLD continued on CNI-based therapy the entire time before PTLD occurred.

In a univariate analysis, gender, multiple organ transplantation, age, type of induction therapy, and rejection were not found to be significantly associated with the diagnosis of PTLD (Table 2). The risk for PTLD increased by 29% for every 10-year increase in the patient age at HT but did not reach statistical significance (*p* = 0.15). The increase in risk of PTLD among patients who received OKT3 as induction therapy compared with those who received ATG or no induction therapy was not significant (HR 1.90, 95% CI: 0.89–4.06; *p* = 0.10). Similarly, rejection was not associated with significantly increased hazard of PTLD (*p* = 0.16, Table 2). A model of those missing EBV status and those with an EBV mismatch showed that having missing EBV status was not associated with PTLD compared to those without EBV mismatch (HR 1.46; 95% CI: 0.63, 2.41; *p* = 0.38). All subsequent analyses of those with missing EBV information were combined with those without EBV mismatch. EBV mismatch was strongly associated with an increased hazard ratio of PTLD when compared to those missing EBV status or without EBV mismatch (HR 10.03, 95% CI: 3.78–26.61; *p* < 0.001). In a univariate analysis including SRL as a time-dependent covariate, SRL was associated with a remarkable decrease in risk of PTLD (HR 0.19, 95% CI: 0.04–0.80; *p* = 0.02) (Table 2).

EBV mismatch and SRL therapy were applied to the stepwise Cox proportional-hazards model. Adjusting for conversion to SRL, EBV mismatch was associated with an over 17-fold increase in hazard of PTLD as compared to those with matched or unknown EBV status (HR 17.6; 95% CI: 6.2–50.3; *p* < 0.001) (Table 3). When adjusting for EBV mismatch status, SRL therapy was found to be associated with an 88% decreased risk of PTLD (HR 0.12, 95% CI: 0.03–0.55; *p* = 0.006). As the main hypothesis of interest was whether the use of SRL affects the risk of PTLD, we investigated the effects of other variables. After jointly adjusting for induction therapy and a history of acute allograft rejection (as time-dependent covariate), as well as for EBV mismatch or any combination of these variables, the use of SRL was independently associated with a decreased risk of PTLD (HRs ranged from 0.12 to 0.20; *p*-values ≤0.03 for all comparisons) (Table 3; models 2–6).

Of the 590 patients who underwent HTs during the study period, 5 patients maintained on CNI therapy were diagnosed with PTLD during the interval 1 to 6 months, for a standardized morbidity ratio (SMR) of 215 (95% CI: 70–501) per 10,000 person years (PY) (Table 4). During the subsequent intervals, the rate of PTLD declined among those who continued on a CNI until it was 90 per 10,000 PY in the interval of 5–10 years post-HT and 64 per 10,000 PY in the last interval after 10 years post-HT. It was not until after 5 years that anyone receiving SRL was diagnosed with PTLD. In the interval of 5–10 years post-HT, one patient developed PTLD for an SMR of 24 (95% CI: 1–134) per 10,000 PY. An additional patient was diagnosed with PTLD while being treated with SRL during the final interval after 10 years post-HT for an SMR of 41 (95% CI: 1–230) per 10,000 PY. Over the entire time frame, HT patients were on SRL-based immunosuppression for a total of 1247 PY and on CNI-based immunosuppression for a total of 3033 PY.

### 3.3. Types of PTLD and Treatment Strategies

Of the 30 patients who developed PTLD in the overall HT cohort, 6 patients (20%) had low-grade lymphoma, whereas 24 patients (80%) developed high-grade lymphoma. Based on histopathologic examinations, EBV was positive in 13 PTLD cases (43.3%). Three patients developed CNS lymphoma and two patients developed cardiac graft lesions consistent with PTLD. The remainder of the patients had lymphoma that involved extranodal sites, including the gastrointestinal tract (*n* = 7), lung (*n* = 2), or soft tissues (*n* = 6), while 10 patients had multiple organ involvement. Treatment of PTLD was as follows: 7 patients (23.3%) had immunosuppression therapy discontinued, 6 patients (20%) received single-agent rituximab, 13 patients (43.3%) received rituximab and chemotherapy, and 4 patients (13.3%) did not receive any treatment. During follow-up, 13 patients (43.3%) achieved complete remission and 13 (43.3%) died; the probability of death was 30.6% at 1 year following PTLD diagnosis.

## 4. Discussion

This study was designed to investigate whether conversion to SRL-based immunosuppression affects the incidence of PTLD following HT in a large double-center HT population. The salient findings of our study are that substitution of CNI by SRL as primary immunosuppression is associated with a substantial decrease in risk of PTLD independent of other known risk factors, including EBV mismatch and type of induction therapy post-HT. Our data are supportive of the protective effects of SRL on PTLD development and suggest that SRL use in lieu of CNI as a maintenance immunosuppression strategy may reduce the risk of PTLD.

Clinical studies are largely limited to the kidney transplant population, reporting a beneficial effect of mTOR inhibitors on PTLD risk in this cohort [15,20]. In one small series of kidney transplant recipients, mTOR inhibitors were used as an adjunct for treatment of PTLD in 19 patients, resulting in a complete remission in 15 patients [15]. However, the impact of mTOR inhibitors on PTLD risk in HT is unclear. We have recently reported on the effect of conversion to SRL on the incidence of all de novo malignancies post-HT in a single institution, with a significant reduction in the risk of malignancies with suggestive greater protection against PTLD found in this cohort [16]. Herein, we expand our previous study by primarily examining the independent, long, double-center experience at Mayo Clinic regarding the impact of conversion to sirolimus on PTLD risk, demonstrating that SRL conversion is specifically associated with a remarkable reduction in the primary outcome of PTLD incidence following HT.

The mechanisms by which SRL (or other mTOR inhibitors) confers protection against PTLD are not understood. A previous study has shown ubiquitous activation of mTOR signaling pathways in malignant lymphoid cells in patients with PTLD, regardless of their EBV genome expression status [21]. In support of this finding, there is a growing body of evidence demonstrating the antiproliferative properties of mTOR inhibitors in PTLD models [12,13,22,23]. Furukawa and colleagues have recently demonstrated that, among patients with PTLD and EBV-positive lymphoma, the PI3K/Akt/mTOR pathway is constitutively active, and that the combination of SRL with PI3K delta inhibitor synergistically suppresses the proliferation of EBV-positive B lymphoma cells [12]. These findings may support a mechanistic explanation of the PTLD risk benefit seen in patients converted to SRL as compared to those maintained on CNI-based immunosuppression in our cohort. Consistent with our results, everolimus, a derivative of sirolimus, has been previously shown to be an effective therapy for patients with untreated diffuse large B-cell lymphoma and for relapsed aggressive lymphoma [24,25].

PTLD is one of the major post-HT malignant diseases, affecting up to 6% of HT recipients, and remains a major issue related to high morbidity and mortality rates [26,27]. PTLD is a significant hindrance to the improvement in long-term survival post-HT. In one study, patients diagnosed with PTLD had an approximately fourfold higher risk of mortality as compared to the matched controls [28]. Multisystem involvement with lymphoma, renal dysfunction, CNS lesions, bone marrow involvement, T-cell subtypes, and older age were identified as factors associated with poor prognosis [1,26,29]. In our cohort, the incidence of PTLD in the CNI group was consistent with previous reports and was significantly lower with the use of SRL. Moreover, mortality among our PTLD cohort was similar to other studies: 30%. Consistent with previous studies, we found that PTLD was associated with decreased survival, with an approximately 2.5-fold increased risk of mortality post-HT [16].

Multiple risk factors for the development of PTLD have been recognized among HT recipients. Among these risk factors, EBV mismatch is one of the strongest predictors of PTLD. Our results are consistent with previous studies showing that EBV mismatch is strongly associated with PTLD, and the majority of our PTLD patients had EBV-positive biopsies [30,31,32,33,34]. OKT3 induction therapy has been shown to be an independent risk factor for PTLD in the HT population [30,31,34], whereas ATG induction therapy showed inconsistent results, with some registry studies showing increased risk of PTLD and others showing no such effect [35,36]. Our data show no significant association of the type of induction therapy with risk of PTLD, nor with recipient age or rejection, despite a trend towards an approximately twofold increase in rates of PTLD with both rejection and OKT3 use as induction therapy. The lack of a significant association between induction therapy and risk of PTLD in our cohort might be explained by the small number of events. However, the low OKT3 dose generally used according to our previous protocol, as well as the transition to ATG induction therapy instead of OKT3 for most of our HT cohort, may have contributed to the attenuated association seen with PTLD events.

Besides the suggestive favorable effects of SRL on PTLD development, early conversion from CNI to SRL has been demonstrated to attenuate CAV progression, improve long-term clinical outcomes after HT, and preserve renal function after CNI withdrawal [7,9,16,37]. The effectiveness of SRL in mitigating CAV is mediated by it inhibitory effects on proliferation and migration of vascular smooth muscle cells [4], but also due to alleviation of T- and B-cell immunity as a result of inhibiting their response to growth factors [5]. The latter might add to the mechanisms underlying the protective effects of SRL against proliferation of abnormal lymphoid cells and PTLD development, as well as against overall de novo malignancies and recurrent non-melanoma skin cancers as shown previously [16]. Therefore, SRL can be potentially used for a multipronged approach to post-HT management for improvement in long-term outcomes.

Our study is limited by its retrospective design and it is therefore subject to the limitations inherent to any study with an observational, retrospective design and non-randomized treatment assignment. Data regarding induction therapy and EBV status could not be obtained for all patients. In addition, doses of immunosuppressive medications used for both induction and maintenance immunosuppression were not recorded for all patients. The small number of PTLD events despite the inclusion of a large cohort of patients and a long follow-up duration is acknowledged. Finally, given the low number of PTLD cases, particularly in the SRL group, we cannot make any conclusions regarding the prognosis of PTLD in the CNI versus the SRL groups. Further studies are needed to understand whether PTLD severity is associated with the type of immunosuppression. Overall, however, the large size of the cohort and the limited data on this subject make this a significant observation. The main strengths of our study are the large cohort size, the length of follow-up, and the standardized and frequently utilized process of conversion to SRL in our institution.

In conclusion, conversion to SRL as primary immunosuppression was associated with a markedly decreased risk of PTLD following HT independent of other known risk factors. The PTLD risk benefit from conversion to SRL was additive to its protective effects on CAV progression and renal function as compared to CNI maintenance therapy. Large prospective studies are required to test whether SRL decreases the development of PTLD in HT recipients, particularly among patients at high PTLD risk.

## Figures and Tables

**Table 1:** Baseline characteristics.

Variable	PTLD	No PTLD	Total
	(*N* = 30)	(*N* = 560)	(*N* = 590)
Age at transplant (years)			
Mean ± SD	53.9 ± 11.1	51.5 ± 11.8	51.6 ± 11.8
Median	56.5	54	54
Male gender (*n*, %)	25 (83.3%)	408 (72.9%)	433 (73.4%)
Multi-organ transplants ^†^ (*n*, %)	3 (10.0%)	60 (10.7%)	63 (10.7%)
EBV mismatch (*N*, *n*, %)	5 of 16 (31%)	13 of 403 (3.2%)	18 of 419 (4.3%)
Induction regimen			
OKT3	19 (86.4%)	191 (46.4%)	210 (48.5%)
ATG	3 (13.6%)	173 (42.1%)	176 (40.7)
Basiliximab	0 (0.0%)	8 (2.0%)	8 (1.8%)
Other	0 (0.0%)	8 (2.0%)	8 (1.8%)
No induction	0 (0.0%)	31 (7.5%)	31 (7.2%)
Missing induction	8	149	157

^†^ 26 had heart transplants plus liver transplants; 33 had heart transplants plus kidney transplants; 4 had heart transplants and both liver and kidney transplants; the remaining 527 had heart transplants alone. ATG, antithymocyte globulin; EBV, Epstein–Barr virus; OKT3, muromonab-CD3; PTLD, post-transplant lymphoproliferative disorder.

**Table 2:** Univariate characteristics associated with increased hazard of PTLD.

Variable	Hazard Ratio	95% CI	*p*-Value
Baseline characteristics			
Mayo Clinic Rochester	1.19	0.40, 3.52	0.76
Patient age at heart transplant per 10 years	1.29	0.91, 1.81	0.15
Male gender	1.58	0.60, 4.14	0.35
Multiple organs transplanted	1.24	0.37, 4.11	0.73
EBV mismatch ^†^	11.87	4.12, 34.19	<0.001
Missing EBV information ^†^	1.46	0.63, 3.41	0.38
EBV mismatch ^‡^	10.03	3.78, 26.61	<0.001
OKT3 induction vs. all others *	1.90	0.89, 4.06	0.10
Time-dependent characteristics			
Sirolimus	0.19	0.04, 0.80	0.02
Rejection	1.71	0.81, 3.60	0.16

^†^ EBV status was considered a mismatch if the donor was EBV-positive and the recipient was EBV-negative. Both EBV mismatches and those missing EBV status were compared to those without an EBV mismatch. ^‡^ EBV status was set considered a mismatch if the donor was EBV-positive and the recipient was EBV-negative. For the purposes of this analysis, anyone without an indication of EBV mismatch was not considered a mismatch. * No induction, antithymocyte globulin (ATG), other induction, or missing induction. EBV, Epstein–Barr virus; OKT3, muromonab-CD3; PTLD, post-transplant lymphoproliferative disorder.

**Table 3:** Multivariable models for association with increased hazard of PTLD.

Model	Variable	Hazard Ratio	95% CI	*p*-Value
1	Stepwise Model *			
	Sirolimus (time-dependent)	0.12	0.03, 0.55	0.006
	EBV mismatch ^‡^	17.65	6.20, 50.25	<0.001
2	Adjusted Models			
	Sirolimus (time-dependent)	0.20	0.05, 0.83	0.03
	Induction **			
	OKT3	1.80	0.84, 3.87	0.13
3	Sirolimus (time-dependent)	0.14	0.03, 0.62	0.009
	EBV mismatch ^‡^	19.43	6.87, 54.97	<0.001
	Induction **			
	OKT3	2.03	0.93, 4.44	0.07
4	Sirolimus (time-dependent)	0.19	0.04, 0.82	0.02
	Rejection (time-dependent)	1.67	0.79, 3.51	0.18
5	Sirolimus (time-dependent)	0.20	0.05, 0.84	0.03
	Rejection (time-dependent)	1.52	0.72, 3.24	0.28
	Induction **			
	OKT3	1.68	0.78, 3.66	0.19
6	Sirolimus (time-dependent)	0.14	0.03, 0.62	0.01
	Rejection (time-dependent)	1.39	0.64, 3.01	0.40
	EBV mismatch ^‡^	18.41	6.51, 52.06	<0.001
	Induction **			
	OKT3	1.89	0.85, 4.20	0.12

* Stepwise Cox proportional hazards with 0.05 to enter and to leave. ^‡^ EBV status was set considered a mismatch if the donor was EBV-positive and the recipient was EBV-negative. All others were not considered a mismatch. ** Listed inductions versus antithymocyte globulin (ATG), other inductions, or no induction. EBV, Epstein–Barr virus; OKT3, muromonab-CD3; PTLD, post-transplant lymphoproliferative disorder.

**Table 4:** Rate of PTLD per 10,000 person years, categorized by Sirolimus treatment.

Time from Heart Transplant		While Not on Sirolimus	While on Sirolimus
0–1 month	Number at risk of PTLD	590	1
	PY during interval	48.42	0.04
	Number with PTLD	0	0
	Rate ^†^	0	0
	95% CI ^†^	(0, 762)	(0, 9624)
1–6 months	Number at risk of PTLD	589	64
	PY during interval	232.68	11.05
	Number with PTLD	5	0
	Rate ^†^	215	0
	95% CI ^†^	(70, 501)	(0, 3338)
6–12 months	Number at risk of PTLD	521	127
	PY during interval	241.19	45.19
	Number with PTLD	30	0
	Rate ^†^	124	0
	95% CI ^†^	(26, 364)	(0, 816)
1–2 years	Number at risk of PTLD	453	167
	PY during interval	374.17	135.93
	Number with PTLD	3	0
	Rate ^†^	76	0
	95% CI ^†^	(16, 222)	(0, 271)
2–5 years	Number at risk of PTLD	420	190
	PY during interval	862.60	396.17
	Number with PTLD	7	0
	Rate ^†^	81	0
	95% CI ^†^	(33, 167)	(0, 93)
5–10 years	Number at risk of PTLD	298	141
	PY during interval	762.62	415.40
	Number with PTLD	7	1
	Rate ^†^	92	24
	95% CI ^†^	(37, 189)	(1, 134)
10+ years	Number at risk of PTLD	138	69
	PY during interval	460.49	242.43
	Number with PTLD	3	1
	Rate ^†^	64	41
	95% CI ^†^	(13, 190)	(1, 230)

^†^ Per 10,000 person years (PY). EBV, Epstein–Barr virus; PTLD, post-transplant lymphoproliferative disorder.

## Data Availability

The data presented in this study are available on request from the corresponding author. The data are not publicly available because they contain information that could compromise the privacy of the study participants.

## Abbreviations

PTLD	post-transplant lymphoproliferative disorder
MTOR	mammalian target of rapamycin
HT	heart transplantation
CNI	calcineurin inhibitor
EBV	Epstein–Barr virus
